# Humanization of the Prostate Microenvironment Reduces Homing of PC3 Prostate Cancer Cells to Human Tissue-Engineered Bone

**DOI:** 10.3390/cancers10110438

**Published:** 2018-11-13

**Authors:** Jacqui A. McGovern, Abbas Shafiee, Ferdinand Wagner, Christoph A. Lahr, Marietta Landgraf, Christoph Meinert, Elizabeth D. Williams, Pamela J. Russell, Judith A. Clements, Daniela Loessner, Boris M. Holzapfel, Gail P. Risbridger, Dietmar W. Hutmacher

**Affiliations:** 1 Centre in Regenerative Medicine, Institute of Health and Biomedical Innovation, Queensland University of Technology (QUT), 60 Musk Avenue, Kelvin Grove, Brisbane, QLD 4059, Australia; jacqui.mcgovern@qut.edu.au (J.A.M.); a.shafiee@qut.edu.au (A.S.); ferdinand.wagner@med.uni-muenchen.de (F.W.); christoph.lahr@hdr.qut.edu.au (C.A.L.); marietta.landgraf@qut.edu.au (M.L.); christoph.meinert@qut.edu.au (C.M.); daniela.lossner@qut.edu.au (D.L.); holzapfel@orthopaedic-oncology.net (B.M.H.); 2 UQ Diamantina Institute, Translational Research Institute, The University of Queensland, Brisbane, QLD 4102, Australia; 3 Department of Pediatric Surgery, Dr. von Hauner Children’s Hospital, Ludwig-Maximilians-University of Munich, Lindwurmstraße 4, 80337 Munich, Germany; 4 Australian Prostate Cancer Research Centre—Queensland, Institute of Health and Biomedical Innovation, School of Biomedical Sciences, Queensland University of Technology, Princess Alexandra Hospital, Translational Research Institute, Brisbane, QLD 4102, Australia; ed.williams@qut.edu.au (E.D.W.); pamela.russell@qut.edu.au (P.J.R.); j.clements@qut.edu.au (J.A.C.); 5 Centre for Cancer and Inflammation, Barts Cancer Institute, Queen Mary University of London, London EC1M 6BQ, UK; 6 Orthopedic Center for Musculoskeletal Research, University of Wuerzburg, Brettreichstraße 11, 97072 Wuerzburg, Germany; 7 Department of Anatomy and Developmental Biology, Biomedicine Discovery Institute, Monash University, Melbourne, VIC 2800, Australia; gail.risbridger@monash.edu; 8 Prostate Cancer Research Program, Cancer Research Division, Peter MacCallum Cancer Centre, Melbourne, VIC 3000, Australia; 9 ARC Industrial Transformation Training Centre In Additive Biomanufacturing, Queensland University of Technology (QUT), 60 Musk Avenue, Kelvin Grove, Brisbane, QLD 4059, Australia

**Keywords:** prostate cancer, bone metastasis, tissue engineering, humanized bone, orthotopic model, cancer-associated fibroblasts, tumor microenvironment

## Abstract

The primary tumor microenvironment is inherently important in prostate cancer (PCa) initiation, growth and metastasis. However, most current PCa animal models are based on the injection of cancer cells into the blood circulation and bypass the first steps of the metastatic cascade, hence failing to investigate the influence of the primary tumor microenvironment on PCa metastasis. Here, we investigated the spontaneous metastasis of PC3 human PCa cells from humanized prostate tissue, containing cancer-associated fibroblasts (CAFs) and prostate lymphatic and blood vessel endothelial cells (BVECs), to humanized tissue-engineered bone constructs (hTEBCs) in NOD-SCID IL2Rγ^null^ (NSG) mice. The hTEBC formed a physiologically mature organ bone which allowed homing of metastatic PCa cells. Humanization of prostate tissue had no significant effect on the tumor burden at the primary site over the 4 weeks following intraprostatic injection, yet reduced the incidence and burden of metastases in the hTEBC. Spontaneous PCa metastases were detected in the lungs and spleen with no significant differences between the humanized and non-humanized prostate groups. A significantly greater metastatic tumor burden was observed in the liver when metastasis occurred from the humanized prostate. Together, our data suggests that the presence of human-derived CAFs and BVECs in the primary PCa microenvironment influences selectively the metastatic and homing behavior of PC3 cells in this model. Our orthotopic and humanized PCa model developed via convergence of cancer research and tissue engineering concepts provides a platform to dissect mechanisms of species-specific PCa bone metastasis and to develop precision medicine strategies.

## 1. Introduction

The complications and high mortality of prostate cancer (PCa) are primarily due to the development of distant metastases [1,2]. Although it is well established that PCa cells frequently metastasize to the bone [3], the causes for its preferential homing to the bone organ are not fully understood. This may be related to limitations of current preclinical PCa metastasis models which frequently fail to recapitulate tumor formation at the orthotopic site, and hence overlook the influence of the cellular and extracellular primary tumor microenvironment on PCa growth and metastatic priming, prompting the development of alternative animal models recapitulating the full metastatic cascade.

Of late, the local tissue microenvironment has gained interest as an important niche for primary and metastatic tumor growth and development [4,5,6]. PCa cells have been shown to interact with the surrounding cellular and extracellular microenvironment in both the local and metastatic milieu [7,8,9]. In particular, human cancer-associated fibroblasts (CAFs), as well as lymphatic and blood vessel endothelial cells (BVECs) have been reported to stimulate the proliferation and metastasis of malignant epithelial cells in vivo [10,11,12,13], indicating the significance of the tumor microenvironment in disease modelling.

We have previously demonstrated the importance of human specific cell-cell interactions within a murine host by generating a humanized tissue-engineered bone construct (hTEBC). The hTEBC was employed as a platform to study the interaction of human cancer cells with a human bone environment following direct intraosseous injection [14,15], or to study species-specific homing to the bone organ following intracardiac injection of cancer cells [16,17]. However, a humanized orthotopic xenograft model of PCa bone metastasis that incorporates the full metastatic cascade has been a quest from a clinical point of view.

Current in vivo mouse models of PCa bone metastasis are typically rooted in cancer cell injection into the blood circulation. This bypasses the first critical steps in the metastatic cascade, where establishment of the primary tumor is followed by local invasion, intravasation of the circulatory system and extravasation to distant metastatic sites, such as bone [1,18]. Furthermore, from a clinical and therapeutic perspective, mouse models of PCa metastasis generally do not account for the important interaction of cancer and stromal cells in the prostate gland or of bone metastases. Therefore, we hypothesized that a preclinical model could be established by applying tissue engineering principles and that this would allow studies of species-specific bone metastasis from cancer cells primed with human stromal cells at the orthotopic tumor site. In the current study, we report spontaneous metastasis of human PC3 cells to a humanized bone from an orthotopic xenograft model of human PCa. This original model provides an important platform to study species-specific metastasis of PCa to the bone and will have important implications in preclinical PCa metastasis studies as well as the development and testing of therapeutic strategies related to personalized medicine.

## 2. Results

### 2.1. Implanted hTEBC Forms an Organ Bone In Immunocompromised Mice

**Figure 1:**
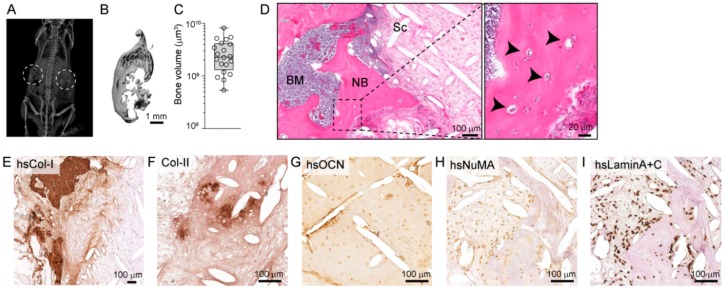
The humanized tissue-engineered bone construct (hTEBC) forms a physiologically representative organ bone in vivo. (**A**) X-ray imaging of the mice 4 weeks after scaffold implantation reveals calcified tissue formation (the dashed circles represent the hTEBC transplantation site). (**B**) Morphology of the hTEBC at the experimental endpoint, 10 weeks after hTEBC implantation and (**C**) quantitative analysis of bone volume from the hTEBC ex vivo using µ-CT analysis (n = 20 hTEBC; individual data points are represented within the box plot). (**D**) Hematoxylin and eosin (H&E) staining for morphological overview of the bone organ depicting bone marrow (BM), newly formed (NB), residual medical grade polycaprolactone (mPCL) scaffold fibers (Sc) and mature osteocytes embedded in the bone matrix (arrowheads). Immunohistochemical staining for (**E**) human-specific type-I collagen (hsCol-I), (**F**) type-II collagen (Col-II), (**G**) human-specific osteocalcin (hsOCN), (**H**) human-specific nuclear mitotic apparatus protein-1 (hsNuMA) and (**I**) human-specific Lamin A+C (hsLaminA+C). Scale bars represent 100 µm and 20 µm.

The organ bone is characterized by the presence of a cortical shell, trabeculae, bone marrow, and bone-resident cells including osteoblasts, osteocytes and osteoclasts, as well as a collagen-rich extracellular matrix (ECM). To establish clinically-relevant models of PCa metastasis to bone incorporating these important hallmarks, we have tissue-engineered a humanized bone organ within an immunocompromised murine host following our previously published protocols [16,17,19,20,21]. First, tubular scaffolds were 3D-printed from medical grade polycaprolactone (mPCL) via melt electrowriting, seeded with human pre-osteoblasts (hOBs) and cultured under osteogenic conditions for 9 weeks to induce osteoblastic differentiation and matrix mineralization (Supplementary Figure S1A). To enable the formation of a human capillary network within the hTEBC, gelatin methacryloyl (GelMA) hydrogels containing human umbilical vein endothelial cells (HUVECs) and bone marrow multipotent mesenchymal stromal cells (MSCs) were prepared as reported previously [20], combined with fibrin glue containing recombinant human bone morphogenetic protein-2 (rhBMP-2), and subcutaneously implanted into the back flanks of NOD-SCID IL2Rγ^null^ (NSG) mice together with the hOB-seeded scaffolds (Supplementary Figure S1B). The formation of mineralized tissue in the hTEBC was confirmed with X-ray imaging 4 weeks after implantation (Figure 1A). At the experimental endpoint, 10 weeks after the hTEBC was implanted, bone was visualized and total bone volume (BV) was quantified using ex vivo µ-CT (Figure 1B,C). The average BV was 2.88 × 10^9^ ± 1.96 × 10^9^ µm^3^ (mean ± SD; n = 20 hTEBC) confirming that bone formation had occurred in the immunocompromised mice.

Evidence of a functional organ bone was further validated by histological analysis of the decalcified hTEBC. Hematoxylin and eosin (H&E) staining revealed that the hTEBC contained newly formed calcified tissue around the mPCL-CaP scaffold fibers, and the bone structure was infiltrated with new bone marrow-like tissue (Figure 1D). Furthermore, the newly-formed bone tissue was embedded with mature osteocytes (Figure 1D). The presence of human ECM was confirmed with immunohistochemical staining for human-specific type-I collagen (hsCol-I; Figure 1E). Bone formation in the implanted hTEBC partially occurred through endochondral ossification as indicated by the presence of type-II collagen (Col-II) within the bone matrix (Figure 1F). The combined expression of human-specific markers for osteocalcin (hsOCN), nuclear mitotic apparatus-1 (hsNuMA) and Lamin A+C (hsLaminA+C) indicated the human origin and therefore survival of the transplanted hOBs in vivo (Figure 1G–I). These results demonstrate that the hTEBC subcutaneously implanted into the murine back flanks generates an organ bone which physiologically recapitulates a localized human bone microenvironment.

### 2.2. Humanization of the Murine Prostate Does Not Influence PC3 Prostate Tumor Growth

CAFs are a prominent cell type present in the cancer stroma, together with BVECs, and are thought to contribute to metastasis from the primary tumor to distant sites, including bone [12,22]. In light of this, we sought to determine if a humanized prostate microenvironment, generated through co-injection of CAFs and BVECs, would stimulate primary tumor growth and metastasis compared to a non-humanized (without CAFs and BVECs) prostate microenvironment. Therefore, following the establishment of the humanized bone niche, we initiated a primary prostate tumor in the NSG mice. PC3 prostate cancer cells expressing luciferase (PC3-luc) were injected into the dorsal lobe of the murine prostate 6 weeks after hTEBC implantation. The non-humanized prostate group received PC3-luc cells only (n = 10 mice), whereas the humanized prostate group received PC3-luc cells co-injected with CAFs and BVECs (n = 10 mice) (Supplementary Figure S1C). One mouse from the humanized group was excluded from the study as it had developed a thymic lymphoma, and an additional 4 mice from each group were excluded from the final analysis as they were sacrificed for ethical reasons before the experimental endpoint. All mice from both groups developed a primary prostate tumor following intraprostatic injection of the PC3-luc cells (100% take-rate).

**Figure 2:**
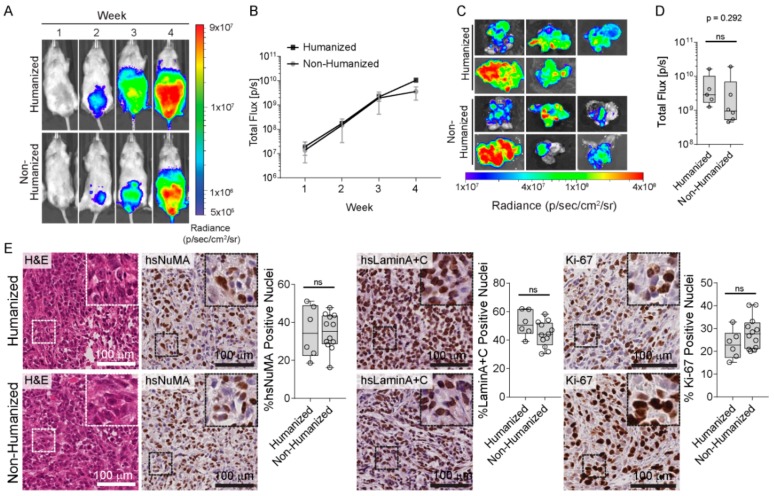
PC3-luc cells form an orthotopic primary prostate tumor in vivo. (**A**) Representative in vivo bioluminescent images (BLI) over 4 weeks following intraprostatic injection. (**B**) Quantification of the in vivo BLI signal in mice with the humanized (black squares) compared to the non-humanized (grey circles) prostate microenvironment (n = 5–6 mice per group; mean ± SEM). (**C**) Ex vivo BLI images of the murine prostate tissues at the 4 weeks experimental endpoint and (**D**) corresponding BLI signals (n = 5–6 prostate tissues; individual data points are shown) demonstrates that there were no significant differences in orthotopic prostate tumor burden between the humanized and non-humanized prostate groups (*p* = 0.292). Data are represented as individual values within the box plot. Statistical analysis was performed using an independent *t*-test. (**E**) Representative histological and immunohistochemical images of the prostate tumors, stained with H&E, hsNuMA, hsLaminA+C and Ki-67. ns: not significant. Scale bars represent 100 µm.

The PC3-luc primary prostate tumor developed over a 4-week time period and was monitored with weekly in vivo bioluminescent imaging (BLI) (Figure 2A). There were no significant differences in primary tumor growth between the humanized and non-humanized prostate groups (Figure 2B). At the experimental endpoint, the primary prostate tumors were harvested and ex vivo BLI signals quantified (Figure 2C,D). There were no significant differences in prostate BLI signal intensity between the humanized prostate (5.38 × 10^9^ ± 6.29 × 10^9^ p/sec; mean ± SEM; n = 5 mice) and non-humanized prostate (4.18 × 10^9^ ± 7.45 × 10^9^ p/sec; mean ± SEM; n = 6 mice) groups (*p* = 0.292; independent *t*-test). These results demonstrate that the presence of the CAFs and BVECs did not influence the relative abundance of the PC3-luc cells within the primary prostate tumor.

The prostatic tissues were further characterized by histological analysis (Figure 2E). H&E staining showed that the prostate tumors in both groups exhibit a highly dedifferentiated morphology, with no obvious morphological differences between the tumors from both groups. Human-specific NuMA and Lamin A+C immunohistochemistry confirmed that a majority of the cells in the prostate tumors were of human origin (Figure 2E). Furthermore, immunostaining for Ki-67, a widely accepted cancer cell proliferation marker [23], indicated that the cells within the prostate tumors were highly proliferative (Figure 2E). However, semi-quantitative image analysis confirmed that there were no differences in human cellular content or proliferative index in response to tumor stroma humanization (Figure 2E). Overall, the inclusion of CAFs and BVECs in the intra-prostatic injection to create a humanized prostate microenvironment did not have a measurable impact on tumor development in the prostate, suggesting that the presence of human stromal cells did not impact on the proliferation of PC3-luc cells within this context.

### 2.3. PC3-Luc Cells Preferentially Metastasize from a Non-Humanized Primary Tumor to a Humanized Bone Organ

**Figure 3:**
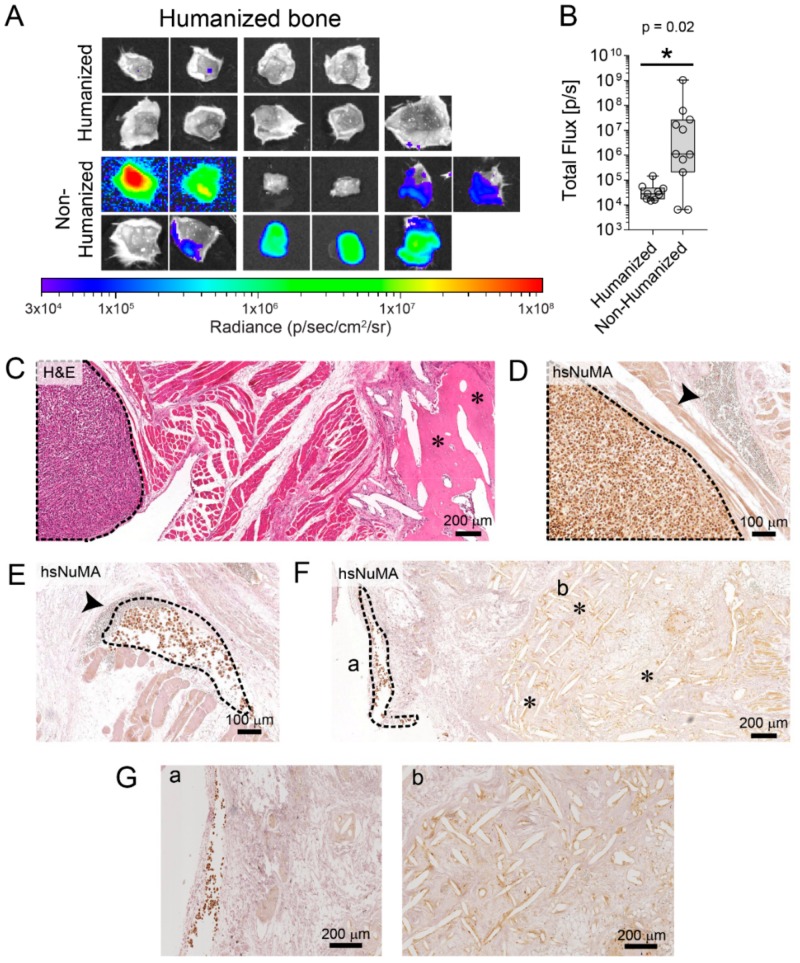
Humanization of the prostate reduces PC3-luc metastasis to the hTEBC. (**A**) Metastasis to the hTEBC (**B**) and corresponding BLI data quantification demonstrates that there was significantly higher tumor burden in the hTEBCs (n = 9–11 scaffolds per group), from the non-humanized prostate compared to the humanized prostate group (data are represented as individual values within box plots. Statistical analysis was performed using a Mann-Whitney U test for non-parametric data). (**C**) Histology (H&E) and (**D**–**G**) immunohistochemical staining for hsNuMA was used to detect PC3-luc metastases in the hTEBCs and the surrounding muscle tissue. PC3-luc cells are outlined with a black dashed line, the hTEBC scaffold fibers are indicated by asterisks (*) and blood vessels are indicated by the arrowhead. (**G**) Represent the high magnification images for part F.

Bone is the most prevalent site of metastatic PCa. In this respect, we examined the dissemination of PC3-luc cells from the primary prostate tumor to the murine skeleton and the hTEBC. The murine bones and the hTEBC were excised at the experimental endpoint, 4 weeks after PC3-luc intraprostatic injection, and analyzed using ex vivo BLI (Figure 3A). We observed metastases in 81.8% of the hTEBC from the non-humanized prostate group, compared to only 22.2% of the hTEBC from the humanized prostate group. Furthermore, there was a significantly greater tumor burden (*p* = 0.02) in the hTEBC after metastasis from the non-humanized prostate tumor (1.05 × 10^8^ ± −3.09 × 10^8^ p/sec; mean ± SEM), compared to the humanized prostate tumor (4.24 × 10^4^ ± 4.08 × 10^4^ p/sec; mean ± SEM; Figure 3B). Interestingly, the average BLI signal in the hTEBC metastases from the humanized prostate group was only slightly higher than the endogenous BLI background level (approximately 2.52 × 10^4^ p/sec), indicating that the overall metastatic burden in the hTEBC was quite low in this group. Histological analysis of the explanted hTEBC revealed PC3-luc metastases present in the surrounding muscle adjacent to the hTEBC (Figure 3C). The human origin of the cancer cells was confirmed with hsNuMA immunohistochemical detection. The cancer cells were frequently observed near surrounding erythrocyte-containing blood vessels, suggesting that the metastases were closely associated with the bone vascular system.

As in human patients, the spine was the site of the murine skeletal system with the highest tumor burden, followed by the hindlimbs and forelimbs (Supplementary Figure S2A–G). There were no significant differences in relative tumor burden within the spine, forelimbs and hindlimbs between the humanized and non-humanized prostate groups, as indicated by ex vivo BLI imaging (*p* = 0.613, *p* = 0.562 and *p* = 0.126, respectively). Altogether these data unexpectedly suggest that the humanized primary tumor reduced metastasis of PC3-luc cells to the humanized bone organ, and also did not influence the metastasis of PCa cells to the murine skeleton.

### 2.4. Metastases of PC3-Luc Cells Were Present in the Lung, Liver, Spleen, Kidneys and the Gastrointestinal (GI) Tract from Both the Humanized and Non-Humanized Prostate Groups

**Figure 4:**
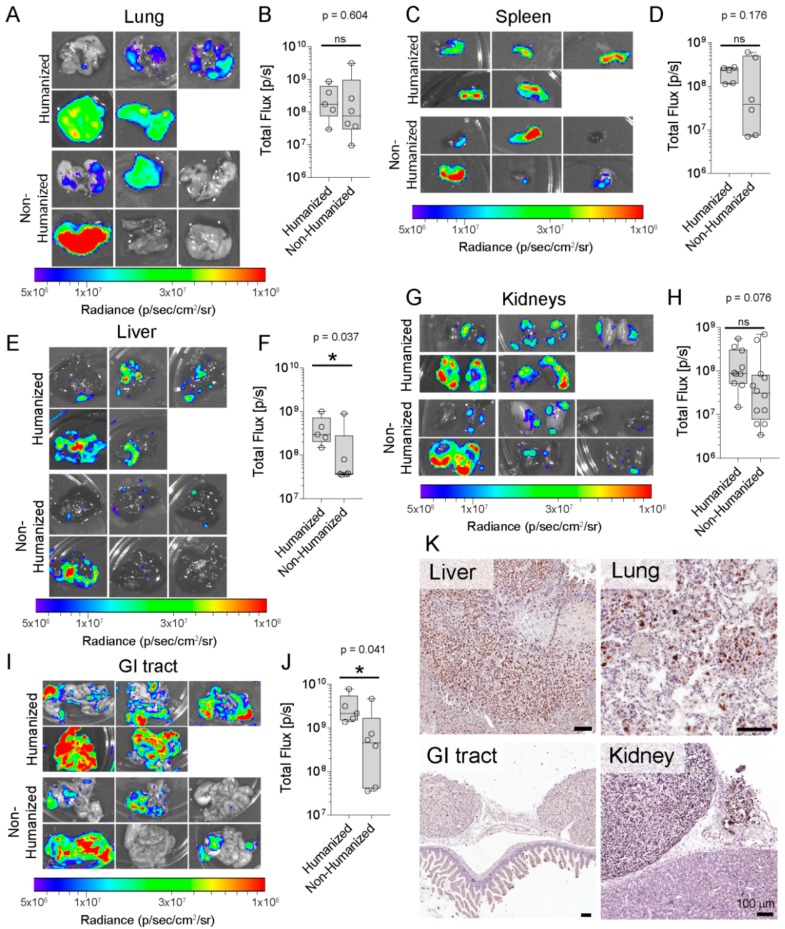
PC3-luc cells metastasized from an orthotopic tumor to the murine organs. (**A**) Ex vivo BLI of metastases to the murine lung (**B**) and IVIS quantification (n = 5–6 lungs per group). The presence of PC3-luc metastases in the murine spleen was detected with ex vivo BLI, (**C**) images of murine spleen and (**D**) corresponding total flux demonstrated no significant differences between both groups (n = 5–6 spleens per group). (**E**) Surface colonization of PC3-luc cells on the murine kidneys and (**F**) quantitative analysis showed no significant differences between groups (n = 10–12 kidneys per group). (**G**) Ex vivo BLI of metastases to the murine liver (**H**) and BLI signal quantification (n = 5–6 livers per group) demonstrate a statistically significant difference in tumor burden between both groups (*p* = 0.037). (**I**) Surface colonization of PC3-luc cells on the murine GI tract and (**J**) quantitative analysis shows significant differences between groups (n = 5–6 GI tracts per group; *p* = 0.041). (**K**) IHC detection of hsNuMA in the murine lung, kidney, liver and GI tract tissue sections. Data are represented as individual values within box plots. Statistical analysis was performed using an independent *t*-test for parametric data, or a Mann-Whitney U test for non-parametric data. Scale bars represent 100 µm. The asterisk (*) represents statistically significant differences between groups (*p* < 0.05) and ns: not significant.

Orthotopic PCa cell inoculation frequently results in soft tissue metastases in murine models [24,25]. Therefore, we investigated the metastases present in other organs and metastatic tumor burden was examined and quantified using ex vivo BLI in the murine lungs, liver, spleen, kidneys and the gastrointestinal (GI) tract (Figure 4). PC3-luc metastases were found in the lungs (*p* = 0.604; Figure 4A,B) and spleen (*p* = 0.176; Figure 4C,D) with no significant difference between both groups. A significantly greater cancer burden was observed in the liver when metastasis had occurred from the humanized prostate (4.33 × 10^8^ ± 1.53 × 10^8^ p/sec) compared to the non-humanized prostate group (1.89 × 10^8^ ± 1.43 × 10^8^ p/sec; *p* = 0.037; Figure 4E,F). The level of kidney PC3-luc colonization was not significantly different between both groups (*p* = 0.076; Figure 4G,H), whereas there was significantly higher tumor burden in the GI tract when the PC3-luc cells had spread from the humanized prostate (3.2 × 10^9^ ± 1.18 × 10^9^ p/sec) compared to the non-humanized prostate group (1.05 × 10^9^ ± 7.2 × 10^8^ p/sec; *p* = 0.041; Figure 4I,J). The human origin of the metastatic foci was confirmed using immunostaining via hsNuMA (Figure 4K). These results confirm that PC3-luc cells metastasize to all organs within NSG mice following intraprostatic injection and that the humanized prostate microenvironment may enhance organ metastasis.

## 3. Discussion

Bone is the most common site of PCa metastasis. Understanding bone-PCa interactions is essential for dissecting the organotrophic homing mechanisms and developing novel therapies [1]. To date, several in vivo models of PCa bone metastasis have been established, including patient-derived xenograft models and more recently, humanized animal models (reviewed by [1,26,27]). Previous works from our team and others have shown that PC3 cells home to an hTEBC in an experimental metastasis scenario [16,28,29]. Implantation of human PCa cells intravenously or directly into the human bone, developed by transplantation of fetal human bone fragments, showed tumors only established in the human bone and not in the murine bones. It was suggested that molecules such as growth factors and their receptors, adhesion molecules, chemotactic factors, proteinases, that regulate bone metastatic cascade are species-specific and contribute in different results in xenograft models of bone metastasis [30,31].

Furthermore, the efficacy of tissue-engineering approaches to study species-specific cancer-bone interactions was investigated in a humanized hematochimeric mouse model of breast cancer metastasis from an orthotopic site to a humanized bone organ [20]. Others have failed to develop tissue-engineered bone construct models for cancer metastasis, as the engineered tissues have only randomly organized mineralized matrix and do not contain a bone marrow, mature matrix embedded osteocytes or human cells [29,32]. Interestingly, Seib et al. (2015) demonstrated metastasis of PC3 cells from an orthotopic site to a TEBC utilizing a BMP-functionalized silk scaffold [33]. In this instance, the randomly organized scaffold architecture was populated with host (murine)-derived cells and did not account for species-specific metastasis as in the current study. Furthermore, the authors manipulated the silk surface to contain receptor activator of nuclear factor K-B ligand (RANKL), which increased PC3 metastasis to the TEBC [33]. In contrast, to our knowledge we are the first to report a model of PCa bone metastasis that recapitulates fully humanized metastasis from a prostate microenvironment to a bone organ.

Recent studies highlight the critical role of stromal cells during primary tumor development and metastasis [5,34]. CAFs are one of the major stromal cell components of the tumor microenvironment and reciprocal feedback loops between CAFs and cancer cells have been suggested. Furthermore, CAFs may induce resistance of cancer cells to therapy [6]. Giannoni et al. (2010) showed the enhancement of tumor growth and development of spontaneous lung metastases when PC3 cells were co-injected subcutaneously with CAFs isolated from PCa patients [35]. Moreover, a role for CAFs in the development of the metastatic niche through remodeling of the tumor microenvironment and secretion of soluble factors has been reported, whereby they promote the metastatic spread of tumor cells and de novo angiogenesis [35,36,37]. Although the impact of CAFs in cancer progression has been shown, their functional contribution to the metastatic process to a humanized bone remains to be investigated. Here, our results showed that humanization of the murine prostate tumor microenvironment, created by co-injecting PC3 prostate cancer cells with CAFs and BVECs, did not influence PC3 primary prostate tumor growth (Figure 2). Unexpectedly, PC3 cells preferentially metastasized from the non-humanized primary tumors to the hTEBC (Figure 3). Furthermore, PC3 cells disseminated to the murine skeleton at the same frequency and intensity from both the humanized and non-humanized prostate microenvironments (Figure S2).

Interestingly, we discovered that PC3 cells metastasized to the soft tissue organ systems, and that liver and GI tract metastases were enhanced from the humanized prostate microenvironment compared to the non-humanized prostate tumors (Figure 4E,F,I,J). There are reports showing that CAFs may promote or inhibit cancer cell metastasis [38,39]. The contradicting results might derive partly from the method used for humanization. Additionally, the differences could be because of the origin of fibroblast and cancer cell types they used. While previous studies employed CAFs or BVECs for humanization, in the current study humanization was performed by co-injection of CAFs and BVECs and PC3 cells. Further investigation is needed to determine the impact of each cell in cancer metastasis. Clearly, these data suggest that the tumor cancer-associated stroma may affect cancer metastasis.

Stromal cells communicate with the surrounding tumor tissue and influence metastasis to distant sites in a phenomenon termed ‘metastatic organotropism’ [26,40,41]. Zhang et al. (2013) reported that breast cancer stroma, rich in CXCL12 and IGF-I secreting mesenchymal cells, selects for a sub-population of cancer cells that have a predisposition to metastasize to bone [22]. Moreover, stromal-produced CXCR4/CXCL12 has been suggested to be crucial for priming breast cancer cells to metastasize to the liver [42]. Co-injection of fibroblasts has been shown to enhance the engraftment of mammary epithelial cells in vivo [11], which contrasts with a previous report that fibroblasts inhibited the growth of malignant breast tissue [43]. Tuxhorn et al. (2002) shed further light on these conflicting results and described the influence of stromal cells co-engrafted with LNCaP PCa cells as patient-specific; some patient-derived stroma enhanced LNCaP tumor formation and host vascular recruitment, while other patient-derived stroma had no measurable influence [7]. Overall, the cancer-associated stroma is important in priming tumor cells for bone metastasis, yet the specific stromal factors which enhance bone organotropism are still to be identified.

Orthotopic models are a cornerstone in in vivo cancer models as they recapitulate most of the physiologically relevant steps of cancer development and metastasis. Experimental metastatic animal models developed by tail-vein injection or intra-cardiac implantation of cancer cells cannot be used as ideal models to investigate the primary tumor or the early steps in the metastatic cascade. To date, numerous orthotopic PCa mouse models have been reported [44,45,46,47]. Intraprostatic implantation of cancer cells allows characterization of molecular and cellular events at the primary tumor and the cross-talk between tumor and microenvironment. However, these models have rarely demonstrated successful bone metastases from the orthotopic niche without serial in vivo passaging [47]. Disseminated cancer cells from murine prostate tumors frequently home to the lymph nodes, lungs and liver [48], as well as to the spleen and kidneys [24,25], similar to our findings (Figure 4C,D,G,H). Here, we injected PC3 cells into the dorsal lobe of the murine prostate to simulate a primary tumor in the native site and to investigate bone metastases. We chose PC3 cells as they were derived from a PCa bone metastasis. We found that the humanized prostate microenvironment did not change primary tumor growth or metastasis to the murine skeleton, but there was preferential metastasis from the non-humanized prostate to the hTEBC. One of the limitations of our model was that the study needed to be terminated 4 weeks after cancer cell injection due to the development of lethal metastases in the murine organs, a known restriction in in vivo bone metastasis studies [49]. Surface colonization, which did not appear to have penetrated into the organ, was detected on the kidneys and the GI tract (Figure 4G,I,K). These organs are located adjacent to the prostate tumor microenvironment and may have been colonized by PC3 cells due to their proximity to the primary prostate tumor. In future studies, we aim to create an orthotopic prostate tumor with a less inherently invasive cell line to recapitulate tumor-stroma interactions over a longer timeframe than 4 weeks. Lastly, understanding the mechanisms involved in reduced homing of PC3 to hTEBC needs further investigation.

## 4. Materials and Methods

### 4.1. Cell Culture

hOBs were isolated from male patients undergoing hip replacement surgery (Queensland University of Technology Research Ethics approval number 1400001024) and cultured as previously described [50]. Luciferase-expressing PC3 cells were maintained in phenol red-free RPMI-1640 (Life Technologies, Mulgrave, VIC, Australia) supplemented with 10% FBS and 100 IU/mL penicillin and 100 µg/mL streptomycin (Life Technologies). Patient-derived prostate cancer associated fibroblasts (CAFs) were isolated as previously described [51] and cultured in phenol red-free RPMI-1640 supplemented with 10% heat-inactivated FBS, 100 IU/mL penicillin and 100 µg/mL streptomycin, 1 nM Testosterone (Sigma, Castle Hill, NSW, Australia) and 10 ng/mL bFGF (Life Technologies). Human prostate-derived CD31^+^ blood vessel endothelial cells (BVECs) were cultured as previously described [13]. Briefly, BVECs were seeded on fibronectin (Sigma)-coated flasks and cultured in the Endothelial Cell Growth Medium 2 Kit (EMG2; PromoCell, Heidelberg, Germany). HUVECs were cultured in EGM2 and MSCs were cultured in MEM-α media containing 20% FBS and 100 IU/mL penicillin and 100 µg/mL streptomycin.

### 4.2. Animal Experiments

All animal experiments were approved by the Queensland University of Technology Animal Ethics Committee (approval number 130000025) in accordance with the Australian Code of Practice for the Care and Use of Animals for Scientific Purposes. Male NOD-*scid* IL2Rγ^null^ (NOD.Cg—*Prkdc^scid^ Il2rg^tm1Wijl^ Hprt^b-m3^*/EshJ; NSG) mice were obtained from the Translational Research Institute in-house breeding colony at 4–6 weeks of age. Animals were maintained under specific pathogen-free and temperature controlled conditions and allowed to acclimatize for 1 week before experimentation. Sterilized food and water were provided ad libitum and mice were kept on a 12 h light-dark cycle. PC3-Luc cells (2.5 × 10^5^) were injected into the murine prostate with or without human CAFs (2 × 10^5^) and human BVECs (5 × 10^4^) in 50 µL of PBS, as previously described [52].

### 4.3. Bioluminescent Imaging (BLI) Analysis

Primary tumor formation and cancer cell metastasis were monitored weekly by in vivo bioluminescent imaging (BLI) using a Xenogen IVIS Spectrum (PerkinElmer, Waltham, MA, USA). Images were acquired 15 min after intraperitoneal injection of 1.5 mg XenoLight d-Luciferin Potassium Salt (PerkinElmer). At the experimental endpoint, 4 weeks after tumor inoculation, the hTEBCs, murine prostate tissue, bones and organs were excised and analyzed using BLI within 20–30 min of D-Luciferin injection. Signals were quantified using the Living Image v4.5.2 software (PerkinElmer) with the manual ROI tool to determine the amount of photons emitted for a given time.

### 4.4. Generation of hTEBC

Tubular mPCL scaffolds were fabricated via melt electrowriting and prepared as described earlier [19,21]. Briefly, the tubular scaffolds were coated with calcium phosphate before seeding with hOBs. The hOB-seeded scaffolds were cultured for 4 weeks in basal hOB media (MEM-α containing 10% FBS and 100 IU/mL penicillin and 100 µg/mL streptomycin), until the scaffolds were fully covered in a dense hOB cell sheet. The scaffolds were then cultured for a further 5 weeks under osteogenic conditions (basal hOB media supplemented with 50 µg/mL l-ascorbic acid-2-phosphate, 0.1 µM dexamethasone and 10 mM β-glycerophosphate; all from Sigma). Formation of a human capillary network within the hTEBC, was performed as described previously [20] (Supplementary Figure S1A).

### 4.5. Histology and Immunohistochemistry

After necropsy, samples were immediately fixed in 4% paraformaldehyde (Sigma) overnight and then transferred to 70% (vol/vol) ethanol until further analysis. Bone samples were decalcified for up to 5 weeks in 10% EDTA (pH 7.4) before embedding in paraffin. Serial sections were used for H&E staining and immunohistochemistry as outlined in Supplementary Table S1. Endogenous peroxidase activity was quenched with 3% hydrogen peroxide (Sigma) for 15 min and non-specific binding sites were blocked with Background Sniper (Biocare Medical, Concord, CA, USA). Primary antibodies were diluted in antibody diluent (Dako, Australia). Positive immunoreactivity was detected with the EnVision+ Dual Link System-HRP Rabbit/Mouse kit (Dako) and was developed with liquid diaminobenzidine chromogen (Dako). Sections were counterstained with Mayer’s Hematoxylin (Sigma) before dehydration and mounting. Human murine tissues were used as positive and negative controls respectively, for human-specific antibodies (Supplementary Figure S3). Images were captured using a Leica SCN400 high-throughput slide scanner.

### 4.6. Statistical Analysis

Graphs were generated using GraphPad Prism v7.01 (GraphPad Software, La Jolla, CA, USA). Statistical data analysis was performed in IBM SPSS Statistics 23 after performing log transformation of the data. Normally distributed data were analyzed using an unpaired t test, whereas data that were not normally distributed were analyzed using a Mann-Whitney U test with a *p* value < 0.05 accepted as significant.

## 5. Conclusions

In conclusion, it was demonstrated that the model developed via convergence of cancer research and tissue engineering concepts provides an important platform to study species-specific metastasis of PCa to the bone and will have eminent implications in dissecting mechanisms of PCa metastasis as well as the development and testing of personalized medicine concepts.

## Supplementary Materials

The following are available online at http://www.mdpi.com/2072-6694/10/11/438/s1, Figure S1: Experimental design, Figure S2: Metastases to the murine skeleton are not influenced by humanization of the primary tumor microenvironment, Figure S3: Validation of human specific antibodies, Table S1: Antibodies and antigen retrieval for immunohistochemistry.

## References

[1] Berish R.B., Ali A.N., Telmer P.G., Ronald J.A., Leong H.S. (2018). Translational models of prostate cancer bone metastasis. Nat. Rev. Urol..

[2] AIHW (2018). Cancer Compendium: Information and Trends by Cancer Type.

[3] Mundy G.R. (2002). Metastasis to bone: Causes, consequences and therapeutic opportunities. Nat. Rev. Cancer.

[4] Langley R.R., Fidler I.J. (2011). The seed and soil hypothesis revisited—The role of tumor-stroma interactions in metastasis to different organs. Int. J. Cancer.

[5] Bussard K.M., Mutkus L., Stumpf K., Gomez-Manzano C., Marini F.C. (2016). Tumor-associated stromal cells as key contributors to the tumor microenvironment. Breast Cancer Res..

[6] Tao L., Huang G., Song H., Chen Y., Chen L. (2017). Cancer associated fibroblasts: An essential role in the tumor microenvironment. Oncol. Lett..

[7] Tuxhorn J.A., McAlhany S.J., Dang T.D., Ayala G.E., Rowley D.R. (2002). Stromal cells promote angiogenesis and growth of human prostate tumors in a differential reactive stroma (DRS) xenograft model. Cancer Res..

[8] Tuxhorn J.A., Ayala G.E., Smith M.J., Smith V.C., Dang T.D., Rowley D.R. (2002). Reactive stroma in human prostate cancer: Induction of myofibroblast phenotype and extracellular matrix remodeling. Clin. Cancer Res..

[9] Taichman R.S., Cooper C., Keller E.T., Pienta K.J., Taichman N.S., McCauley L.K. (2002). Use of the stromal cell-derived factor-1/CXCR4 pathway in prostate cancer metastasis to bone. Cancer Res..

[10] Da J., Lu M., Wang Z. (2015). Estrogen Receptor Alpha (ERalpha)-Associated Fibroblasts Promote Cell Growth in Prostate Cancer. Cell Biochem. Biophys..

[11] Kuperwasser C., Chavarria T., Wu M., Magrane G., Gray J.W., Carey L., Richardson A., Weinberg R.A. (2004). Reconstruction of functionally normal and malignant human breast tissues in mice. Proc. Natl. Acad. Sci. USA.

[12] Olumi A.F., Grossfeld G.D., Hayward S.W., Carroll P.R., Tlsty T.D., Cunha G.R. (1999). Carcinoma-associated fibroblasts direct tumor progression of initiated human prostatic epithelium. Cancer Res..

[13] Zeng Y., Opeskin K., Goad J., Williams E.D. (2006). Tumor-induced activation of lymphatic endothelial cells via vascular endothelial growth factor receptor-2 is critical for prostate cancer lymphatic metastasis. Cancer Res..

[14] Hesami P., Holzapfel B.M., Taubenberger A., Roudier M., Fazli L., Sieh S., Thibaudeau L., Gregory L.S., Hutmacher D.W., Clements J.A. (2014). A humanized tissue-engineered in vivo model to dissect interactions between human prostate cancer cells and human bone. Clin. Exp. Metastasis..

[15] Quent V., Taubenberger A.V., Reichert J.C., Martine L.C., Clements J.A., Hutmacher D.W., Loessner D. (2018). A humanised tissue-engineered bone model allows species-specific breast cancer-related bone metastasis in vivo. J. Tissue Eng. Regen. Med..

[16] Holzapfel B.M., Wagner F., Loessner D., Holzapfel N.P., Thibaudeau L., Crawford R., Ling M.T., Clements J.A., Russell P.J., Hutmacher D.W. (2014). Species-specific homing mechanisms of human prostate cancer metastasis in tissue engineered bone. Biomaterials.

[17] Thibaudeau L., Taubenberger A.V., Holzapfel B.M., Quent V.M., Fuehrmann T., Hesami P., Brown T.D., Dalton P.D., Power C.A., Hollier B.G. (2014). A tissue-engineered humanized xenograft model of human breast cancer metastasis to bone. Dis. Model. Mech..

[18] Simmons J.K., Hildreth B.E., Supsavhad W., Elshafae S.M., Hassan B.B., Dirksen W.P., Toribio R.E., Rosol T.J. (2015). Animal Models of Bone Metastasis. Vet. Pathol..

[19] Martine L.C., Holzapfel B.M., McGovern J.A., Wagner F., Quent V.M., Hesami P., Wunner F.M., Vaquette C., De-Juan-Pardo E.M., Brown T.D. (2017). Engineering a humanized bone organ model in mice to study bone metastases. Nat. Protoc..

[20] Shafiee A., McGovern J.A., Lahr C.A., Meinert C., Moi D., Wagner F., Landgraf M., De-Juan-Pardo E., Mazzieri R., Hutmacher D.W. (2018). Immune system augmentation via humanization using stem/progenitor cells and bioengineering in a breast cancer model study. Int. J. Cancer.

[21] Wagner F., Holzapfel B.M., McGovern J.A., Shafiee A., Baldwin J.G., Martine L.C., Lahr C.A., Wunner F.M., Friis T., Bas O. (2018). Humanization of bone and bone marrow in an orthotopic site reveals new potential therapeutic targets in osteosarcoma. Biomaterials.

[22] Zhang X.H., Jin X., Malladi S., Zou Y., Wen Y.H., Brogi E., Smid M., Foekens J.A., Massague J. (2013). Selection of bone metastasis seeds by mesenchymal signals in the primary tumor stroma. Cell.

[23] Thakur S.S., Li H., Chan A.M.Y., Tudor R., Bigras G., Morris D., Enwere E.K., Yang H. (2018). The use of automated Ki67 analysis to predict Oncotype DX risk-of-recurrence categories in early-stage breast cancer. PLoS ONE.

[24] Bastide C., Bagnis C., Mannoni P., Hassoun J., Bladou F. (2002). A Nod Scid mouse model to study human prostate cancer. Prostate Cancer Prostatic Dis..

[25] Nandana S., Tripathi M., Duan P., Chu C.Y., Mishra R., Liu C., Jin R., Yamashita H., Zayzafoon M., Bhowmick N.A. (2017). Bone Metastasis of Prostate Cancer Can Be Therapeutically Targeted at the TBX2-WNT Signaling Axis. Cancer Res..

[26] Shafiee A., Hutmacher D.W. (2018). Modelomics to Investigate Cancer Bone Metastasis. Curr. Mol. Biol. Rep..

[27] McGovern J.A., Griffin M., Hutmacher D.W. (2018). Animal models for bone tissue engineering and modelling disease. Dis. Model. Mech..

[28] Bersani F., Lee J., Yu M., Morris R., Desai R., Ramaswamy S., Toner M., Haber D.A., Parekkadan B. (2014). Bioengineered implantable scaffolds as a tool to study stromal-derived factors in metastatic cancer models. Cancer Res..

[29] Moreau J.E., Anderson K., Mauney J.R., Nguyen T., Kaplan D.L., Rosenblatt M. (2007). Tissue-engineered bone serves as a target for metastasis of human breast cancer in a mouse model. Cancer Res..

[30] Nemeth J.A., Harb J.F., Barroso U., He Z., Grignon D.J., Cher M.L. (1999). Severe combined immunodeficient-hu model of human prostate cancer metastasis to human bone. Cancer Res..

[31] Bissell M.J., Le Beyec J., Anderson R.L. (2002). Prostate cancer in bone: Importance of context for inhibition of matrix metalloproteinases. J. Natl. Cancer Inst..

[32] Schuster J., Zhang J., Longo M. (2006). A novel human osteoblast-derived severe combined immunodeficiency mouse model of bone metastasis. J. Neurosurg. Spine.

[33] Seib F.P., Berry J.E., Shiozawa Y., Taichman R.S., Kaplan D.L. (2015). Tissue engineering a surrogate niche for metastatic cancer cells. Biomaterials.

[34] Quail D.F., Joyce J.A. (2013). Microenvironmental regulation of tumor progression and metastasis. Nat. Med..

[35] Giannoni E., Bianchini F., Masieri L., Serni S., Torre E., Calorini L., Chiarugi P. (2010). Reciprocal activation of prostate cancer cells and cancer-associated fibroblasts stimulates epithelial-mesenchymal transition and cancer stemness. Cancer Res..

[36] Cirri P., Chiarugi P. (2011). Cancer associated fibroblasts: The dark side of the coin. Am. J. Cancer Res..

[37] Erez N., Truitt M., Olson P., Arron S.T., Hanahan D. (2010). Cancer-Associated Fibroblasts Are Activated in Incipient Neoplasia to Orchestrate Tumor-Promoting Inflammation in an NF-kappaB-Dependent Manner. Cancer Cell.

[38] Shintani Y., Abulaiti A., Kimura T., Funaki S., Nakagiri T., Inoue M., Sawabata N., Minami M., Morii E., Okumura M. (2013). Pulmonary fibroblasts induce epithelial mesenchymal transition and some characteristics of stem cells in non-small cell lung cancer. Ann. Thoracic Surg..

[39] Wang L., Cao L., Wang H., Liu B., Zhang Q., Meng Z., Wu X., Zhou Q., Xu K. (2017). Cancer-associated fibroblasts enhance metastatic potential of lung cancer cells through IL-6/STAT3 signaling pathway. Oncotarget.

[40] Chen W., Hoffmann A.D., Liu H., Liu X. (2018). Organotropism: New insights into molecular mechanisms of breast cancer metastasis. NPJ Precis. Oncol..

[41] Lu X., Kang Y. (2007). Organotropism of breast cancer metastasis. J. Mammary Gland Biol. Neoplasia.

[42] Wendel C., Hemping-Bovenkerk A., Krasnyanska J., Mees S.T., Kochetkova M., Stoeppeler S., Haier J. (2012). CXCR4/CXCL12 participate in extravasation of metastasizing breast cancer cells within the liver in a rat model. PLoS ONE.

[43] Zhang X., Claerhout S., Prat A., Dobrolecki L.E., Petrovic I., Lai Q., Landis M.D., Wiechmann L., Schiff R., Giuliano M. (2013). A renewable tissue resource of phenotypically stable, biologically and ethnically diverse, patient-derived human breast cancer xenograft models. Cancer Res..

[44] Pavese J., Ogden I.M., Bergan R.C. (2013). An orthotopic murine model of human prostate cancer metastasis. J. Vis. Exp..

[45] Cifuentes F.F., Valenzuela R.H., Contreras H.R., Castellon E.A. (2015). Development of an orthotopic model of human metastatic prostate cancer in the NOD-SCIDgamma mouse (Mus musculus) anterior prostate. Oncol. Lett..

[46] Shahryari V., Nip H., Saini S., Dar A.A., Yamamura S., Mitsui Y., Colden M., Bucay N., Tabatabai L.Z., Greene K. (2016). Pre-clinical Orthotopic Murine Model of Human Prostate Cancer. J. Vis. Exp..

[47] Yang M., Jiang P., Sun F.X., Hasegawa S., Baranov E., Chishima T., Shimada H., Moossa A.R., Hoffman R.M. (1999). A fluorescent orthotopic bone metastasis model of human prostate cancer. Cancer Res..

[48] Penet M.F., Pathak A.P., Raman V., Ballesteros P., Artemov D., Bhujwalla Z.M. (2009). Noninvasive multiparametric imaging of metastasis-permissive microenvironments in a human prostate cancer xenograft. Cancer Res..

[49] Kuchimaru T., Kataoka N., Nakagawa K., Isozaki T., Miyabara H., Minegishi M., Kadonosono T., Kizaka-Kondoh S. (2018). A reliable murine model of bone metastasis by injecting cancer cells through caudal arteries. Nat. Commun..

[50] Reichert J.C., Quent V.M., Burke L.J., Stansfield S.H., Clements J.A., Hutmacher D.W. (2010). Mineralized human primary osteoblast matrices as a model system to analyse interactions of prostate cancer cells with the bone microenvironment. Biomaterials.

[51] Taylor R.A., Toivanen R., Frydenberg M., Pedersen J., Harewood L., Australian Prostate Cancer B., Collins A.T., Maitland N.J., Risbridger G.P. (2012). Human epithelial basal cells are cells of origin of prostate cancer, independent of CD133 status. Stem Cells.

[52] Park S.I., Kim S.J., McCauley L.K., Gallick G.E. (2010). Pre-clinical mouse models of human prostate cancer and their utility in drug discovery. Curr. Protoc. Pharmacol..

