# Supplementary material for: Humanization of the Prostate Microenvironment Reduces Homing of PC3 Prostate Cancer Cells to Human Tissue-Engineered Bone
Source: Cancers (Basel). 2018 Nov 13;10(11):438. doi: 10.3390/cancers10110438 (PMC6265886; doi:10.3390/cancers10110438)
Supplement: Supplementary file 1 [file cancers-10-00438-s001.pdf]

Supplementary Materials: Humanization of the Prostate Microenvironment Reduces Homing of PC3 Prostate Cancer Cells to Human Tissue-Engineered Bone

Jacqui A. McGovern, Abbas Shafiee, Ferdinand Wagner, Christoph A. Lahr, Marietta Landgraf, Christoph Meinert, Elizabeth D. Williams, Pamela J. Russell, Judith A. Clements, Daniela Loessner, Boris M. Holzapfel, Gail P. Risbridger and Dietmar W. Hutmacher

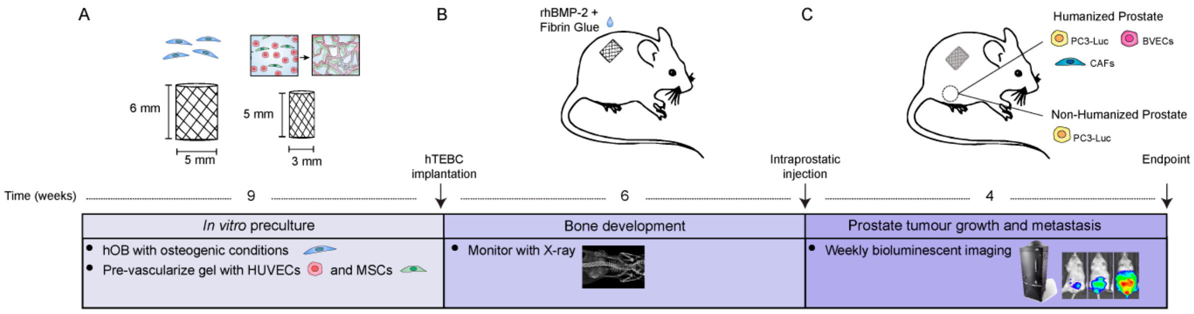

Figure S1. Experimental design. (**A**) The human tissue-engineered bone construct (hTEBC) was created by seeding human osteoblasts (hOB) onto tubular, calcium phosphate (CaP)-coated melt electrospun medical grade polycaprolactone (mPCL) scaffolds. Once the hOBs formed a dense cell and ECM network throughout the scaffold architecture, culture conditions were switched to osteogenic media for 9 weeks total culture. One week before implantation GelMA hydrogels were prepared containing bone marrow MSCs-GFP and HUVECs-mCherry and cultured to form capillary-like networks. The GelMA hydrogels were inserted into the lumen of the hTEBC and combined with recombinant human bone morphogenetic protein-2 (rhBMP-2) and fibrin glue prior to implantation. (**B**) The bone was allowed to develop for 6 weeks before intraprostatic injection. (**C**) Humanization of the murine prostate was performed via intraprostatic injection of PC3-luc cells together with CAFs and BVECs, whereas the non-humanized group received PC3-luc cells only.

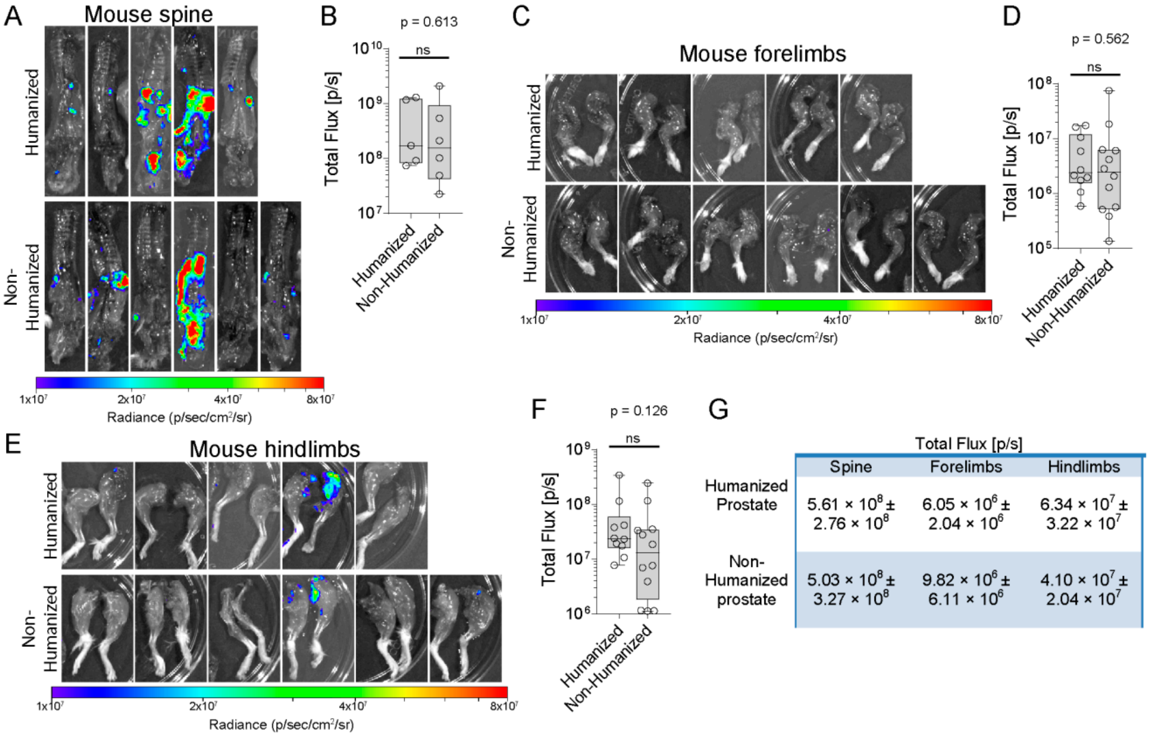

	Total Flux [p/s]		
	Spine	Forelimbs	Hindlimbs
Humanized Prostate	$5.61 \times 10^8 \pm 2.76 \times 10^8$	$6.05 \times 10^6 \pm 2.04 \times 10^6$	$6.34 \times 10^7 \pm 3.22 \times 10^7$
Non-Humanized prostate	$5.03 \times 10^8 \pm 3.27 \times 10^8$	$9.82 \times 10^6 \pm 6.11 \times 10^6$	$4.10 \times 10^7 \pm 2.04 \times 10^7$

Figure S2. Metastases to the murine skeleton are not influenced by humanization of the primary tumor microenvironment. (**A**) Ex vivo BLI of metastases to the murine spine (**B**) and quantification (n = 5–6 spines per group). The presence of PC3-luc metastases in the mouse limbs was detected with ex vivo BLI, (**C**) images of murine forelimbs and (**D**) corresponding total flux demonstrated no significant differences between both groups (n = 10–12 limbs per group). (**E**) Metastases in the murine hindlimbs and (**F**) quantitative analysis suggested no significant differences between groups (n = 10–12 limbs per group). Data are represented as individual values within box plots, depicting the upper and lower quartiles, median, minimum and maximum. (**G**) Summary of the total flux (p/s) of the murine spine, forelimbs and hindlimbs (mean ± SEM) within the humanized and non-humanized prostate groups. Statistical analysis was performed using an independent t-test for parametric data, or a Mann-Whitney U test for non-parametric data.

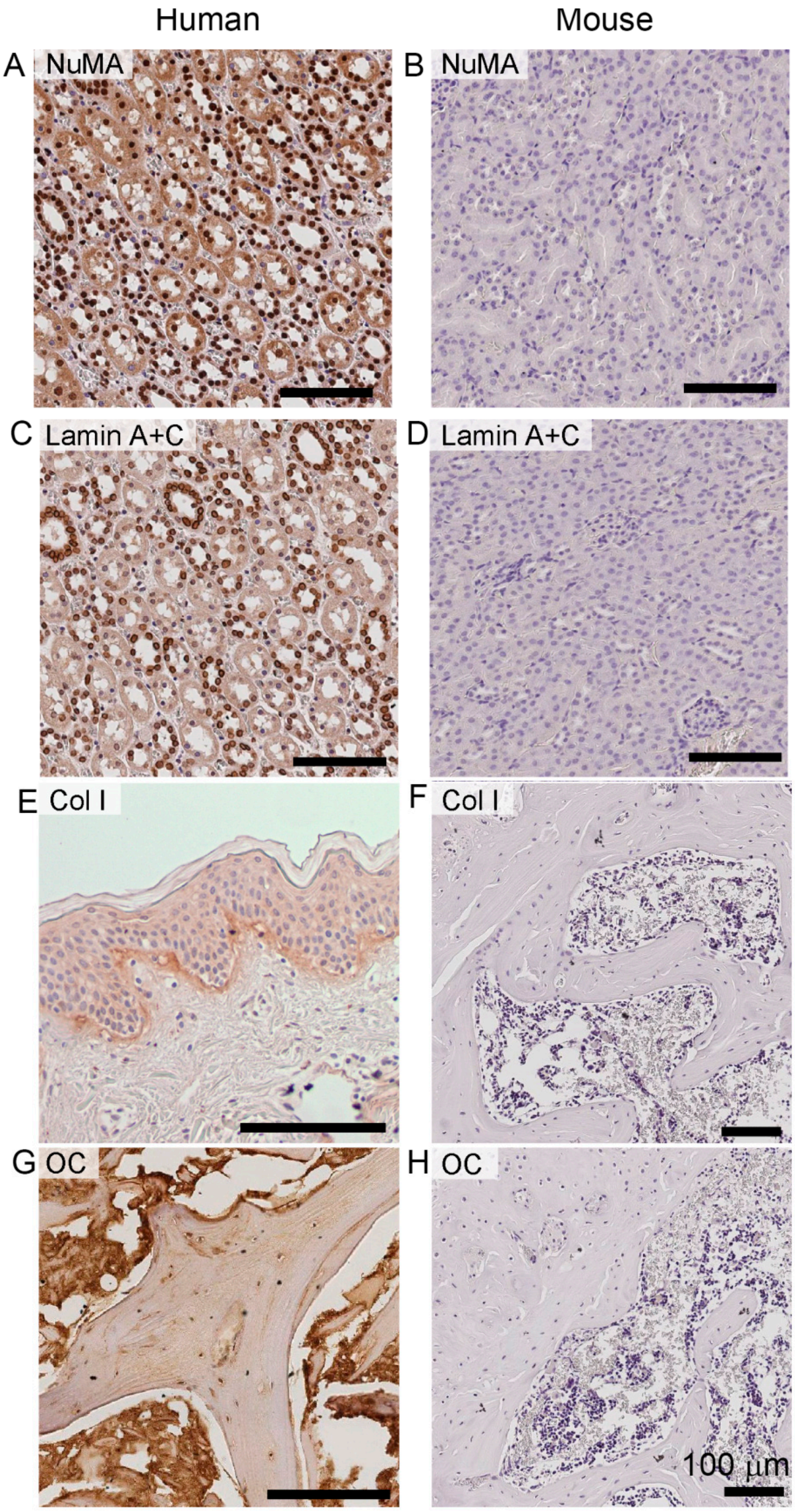

Figure S3. Validation of human specific antibodies. An anti-NuMA antibody was used to probe (**A**) human kidney or (**B**) murine kidney tissue sections. An anti-Lamin A+C antibody was applied to (**C**) human kidney and (**D**) murine kidney tissue sections. (**E**) Human skin and (**F)** murine bone were probed using an anti-Col-I antibody. An anti-OC antibody was applied to (**G**) human bone and (**H**) murine bone. Positive immunoreactivity is indicated by the brown staining. Tissues were counter-stained blue with hematoxylin. The scale bars represent 100 µm.

Table S1. Antibodies and antigen retrieval for immunohistochemistry.

Antibody	Company	Product code	Antigen Retrieval	Incubation
Human-specific Lamin A+C	Abcam	ab108595	Tris-EDTA Buffer+0.1% Tween-20, pH 9.0 (95 °C/5 min)	1:300, 1 h at RT
Human-specific NuMA	Abcam	ab97585	Tri-sodium Citrate Buffer+0.1% Tween-20, pH 6.0 (95 °C/5 min)	1:300, 1 h at RT
Human-specific type I collagen	Abcam	ab23446	Proteinase K, 15 min at RT	1:100, overnight at 4 °C
Type II collagen	DSHB	II-II6B3	Proteinase K, 15 min at RT	1:200, overnight at 4 °C
Human-specific osteocalcin	Abcam	ab13420	Proteinase K, 15 min at RT	1:200, overnight at 4 °C
